# Cytidine 5′-Diphosphocholine (Citicoline): Evidence for a Neuroprotective Role in Glaucoma

**DOI:** 10.3390/nu12030793

**Published:** 2020-03-18

**Authors:** Stefano Gandolfi, Giorgio Marchini, Aldo Caporossi, Gianluca Scuderi, Livia Tomasso, Andrea Brunoro

**Affiliations:** 1 Ophthalmology Unit, Department of Biological, Biotechnological and Translational Sciences, University of Parma, Via Gramsci, 14, 43126 Parma, Italy; stefano.gandolfi@unipr.it; 2 Ophthalmology Unit, Department of Neurosciences, Biomedicine and Movement, University of Verona, P. le L. A. Scuro, 10, 37134 Verona, Italy; giorgio.marchini@univr.it; 3 Ophthalmology Unit, Catholic University of the Sacred Heart, Fondazione Policlinico Universitario A. Gemelli, Rome, Italy., Largo F. Vito 1, 00168 Rome, Italy; aldo.caporossi@unicatt.it; 4 Ophthalmology Unit, St. Andrea Hospital, NESMOS Department, University of Rome “Sapienza”, Via di Grottarossa 1035/1039, 00189 Rome, Italy; gianluca.scuderi@uniroma1.it; 5 Bausch & Lomb IOM spa Viale Martesana 12, 20090 Vimodrone (MI), Italy; livia.tomasso@bausch.com

**Keywords:** citicoline, glaucoma, neurodegeneration, neuroprotection, retinal ganglion cells, supplementation

## Abstract

Glaucoma, a heterogeneous set of progressively degenerative optic neuropathies characterized by a loss of retinal ganglion cells (RGCs) and typical visual field deficits that can progress to blindness, is a neurodegenerative disease involving both ocular and visual brain structures. Although elevated intraocular pressure (IOP) remains the most important modifiable risk factor of primary open-angle glaucoma (POAG) and is the main therapeutic target in treating glaucoma, other factors that influence the disease course are involved and reaching the optimal IOP target does not stop the progression of glaucoma, as the visual field continues to narrow. In addition to a managed IOP, neuroprotection may be beneficial by slowing the progression of glaucoma and improving the visual defects. Citicoline (cytidine 5′-diphosphocholine) is a naturally occurring endogenous compound that has been investigated as a novel therapeutic agent for the management of glaucoma. Citicoline has demonstrated activity in a range of central neurodegenerative diseases, and experimental evidence suggests a it performs a neuromodulator and neuroprotective role on neuronal cells, including RGCs, associated with improvement in visual function, extension of the visual field and central benefits for the patient. This review aims to critically summarize the current evidence for the neuroprotective properties of citicoline in glaucoma.

## 1. Introduction

Glaucoma is a heterogeneous set of progressively degenerative optic neuropathies with common characteristics that include a loss of retinal ganglion cells (RGCs) and typical visual field deficits that can progress to blindness [1,2]. 

The global prevalence of the most common form, primary open-angle glaucoma (POAG), is approximately 3.1%, compared with about 0.5% for primary angle-closure glaucoma (PACG) [3]. Men are somewhat more likely to have POAG than women, as are people of African ancestry compared with Europeans [3]. The prevalence of POAG is highest in Africa (4.2%), whereas PACG is most prevalent in Asia (1.1%). The burden of glaucoma is global; data from robust population-based prevalence models predict that there will be between 76 and 79 million people aged over 40 years affected by glaucoma by 2020 and 112 million by 2040, partly explained by the rapid increase in the aging population worldwide [3]. 

Glaucoma is a leading cause of irreversible blindness worldwide [1,4,5]. Globally, in 2010, glaucoma was the cause of blindness in 2.1 million (6.6%) of the 32.4 million blind individuals in the world and caused moderate-to-severe visual impairment in 4.2 million of the 191 million vision-impaired adults [5]. 

Although elevated intraocular pressure (IOP), also known as ocular hypertension, is considered to be the most important modifiable risk factor of POAG and remains the main therapeutic target in treating glaucoma, other factors that may modify the course of the disease are involved, and controlling IOP does not stop the progression of glaucoma [6,7]. For instance, a retrospective study conducted at the Ophthalmology Department of a referral University Hospital in Sweden revealed that, after a median duration from diagnosis of 12 years, monocular blindness occurred in 42.2% and binocular blindness in 16.4% of the 592 patients who died between 2006 and 2010 [8]. Analysis of long-term trends in glaucoma-related vision loss suggests that risk factors other than IOP are important in the pathogenesis of glaucoma-related neuronal damage [8,9]. 

Undetectable or asymptomatic disease is a major part of the glaucoma continuum, and a substantial proportion of patients with glaucoma remain undiagnosed [10]. Therefore, early detection during routine ophthalmological examinations is important to identify at-risk subjects before symptoms manifest [1], at which point timely initiation of effective interventions before vision loss progresses is an unmet need, as pharmacological treatment is at present primarily reserved for patients with symptomatic glaucoma [6]. As a result, glaucomatous optic neuropathy and visual impairment are already present once the treatment is initiated. Current treatment approaches consist of pharmacological therapy, laser, and surgical procedures, which aim to reduce elevated IOP to a level that lessens further optic nerve damage, but which do not completely stop the progression of visual damage [1,11]. Topical prostaglandin analogs (latanoprost, tafluprost, travoprost, etc.) are typically considered as first-choice medical treatments for lowering IOP due to once-daily dosing and favorable systemic side effects profile; second-line agents include topical β-blockers (timolol, betaxolol, levobunolol, etc.), carbonic anhydrase inhibitors (dorzolamide, brinzolamide), selective α2-adrenergic antagonists (brimonidine, apraclonidine) and topical cholinergic agonists (carbachol, pilocarpine) [1]. These medications lower IOP by decreasing aqueous production, reducing outflow resistance, or a combination of both.

A number of novel therapeutics for the management of glaucoma have been investigated or are under investigation. These include purinergic ligands, KATP channel activators, PI3K/Akt activators, glutamate antagonists, antioxidants, gases (including nitric oxide, carbon monoxide, and hydrogen sulfide), nitric oxide synthase inhibitors, non-glucocorticoid steroidal compounds, cannabinoids, dopamine and serotonin receptors ligands, neurotrophic factors, histone deacetylase inhibitors, small interference RNA, Rho-kinase inhibitors, and citicoline [12,13]. Of these, Rho-kinase inhibitors and NO-donating agents have recently been approved in glaucoma. On the other hand, citicoline (cytidine 5′-diphosphocholine)—a naturally occurring (endogenous) compound—has been studied in ophthalmology for 30 years, and is supported by evidence from in vitro, in vivo, animal, clinical and randomized controlled trials in the published literature. Citicoline, which is metabolized in the body to cytidine and choline, is active in the biosynthetic pathway of cell membrane phospholipids and it is able to increase levels of neurotransmitters in the central nervous system (CNS) [11,14]. Citicoline has shown efficacy in the treatment of cognitive and behavioral disturbances in elderly patients with chronic cerebral disorders and it has shown activity in stroke, Alzheimer’s disease, Parkinson’s disease, and traumatic brain injury [14].

This paper will review the evidence for the neuroprotective properties of citicoline in glaucoma.

## 2. Pathophysiology of Glaucoma 

Glaucoma is a complex, multifactorial degenerative optic neuropathy characterized by RGC death and irreversible peripheral and central visual field vision loss [1,2]. An in-depth discussion of the pathophysiology of glaucoma is beyond the scope of this article, and the understanding of the pathogenesis of glaucoma and the factors contributing to its progression remain incomplete [2]. Although the level of IOP is related to RGC death, nerve damage may continue even when pressure is monitored and treated; glaucomatous optic neuropathy is evident also in individuals with IOPs within the normal range [2]. 

In animal models with unilateral experimentally-induced glaucoma, central neuronal damage is limited to the side of the glaucomatous eye [15,16]. The impairment of the innermost retinal layers may appear early in the natural history of glaucoma, preceding the onset of visual field defects [17], and followed by transsynaptic degeneration-related impairment in postretinal visual pathways, specifically at the level of the lateral geniculate nucleus [15,18,19,20]. Therefore, on the basis of experimental models, it is proposed that glaucoma can be considered a disease involving not only the ocular structures but also of compromised integrity of visual brain structures.

It has been demonstrated in animal models of glaucoma, that the number and volume of relay neurons, responsible for projecting to the visual cortex, were reduced only in the lateral geniculate nucleus of the glaucoma-affected eye [16,18]. Although an elevated IOP was associated with the size, density, and the number of neurons in the lateral geniculate nucleus, damage occurred even with moderate levels of intraocular hypertension [18]. Degenerative changes in the lateral geniculate nucleus, atrophy of the intracranial optic nerve, and thinning of the visual cortex have also been observed in humans, demonstrating that central neural alterations are closely related to disease progression and optic papilla damage [21].

Nevertheless, evidence from the animal models suggests that the death of the RGCs is the crucial pathological event in glaucoma: intraocular hypertension cause stress and strain on posterior structures of the eye, resulting in compression- and deformation-related mechanical damage to the axons of RGCs and disruption of axonal transport [1]. The damaged RGCs are in turn responsible for secondary degeneration of adjacent neuronal cells in the extracellular microenvironment via a process of trans-synaptic degeneration, which occurs early in the course of glaucoma, and it is not associated with elevated IOP only [11,22,23]. Regardless of the primary insult, the ultimate pathway is a mitochondrial-mediated programmed cell death (apoptosis) process resulting in accelerated death of the RGCs [11,23] Retinal ganglion cell apoptosis is recognized as an early manifestation of cell death in glaucoma, and a study in patients with glaucoma found that up to 36% of RGCs may be lost before normal field tests are able to discriminate visual field defects [24].

As discussed, elevated IOP alone does not fully explain RGC loss, and a number of events may converge to induce RGC degeneration, including impaired microcirculation, ischemia/reperfusion damage, oxidative and/or nitrosative stress, neurotrophic growth factor deprivation, mitochondrial dysfunction, activation of autoimmunity, and glutamate-induced neurotoxicity (glutamate excitotoxicity) as part of the sequence of pathological events leading to optic neuropathy [2,6,11,25,26,27,28].

The expanding knowledge of the etiopathogenesis of glaucoma and the need for an effective early treatment to stop the progression of visual defects have stimulated research into new therapeutic approaches.

## 3. Neuroprotection in Glaucoma

**Figure 1:**
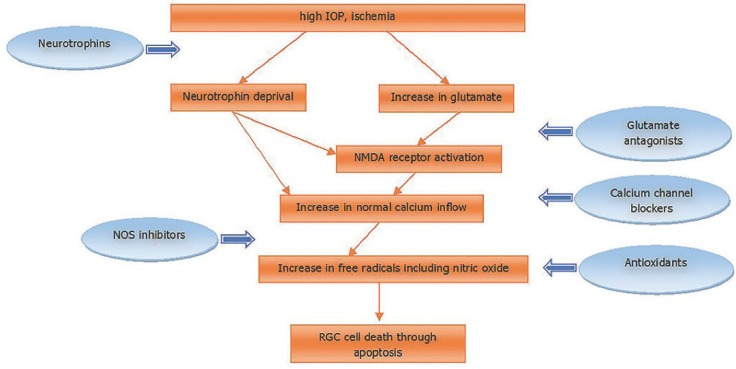
Proposed pathway of retinal ganglion cell (RGC) death and presumed mechanisms of action of neuroprotective agents. IOP, intraocular pressure; NMDA, n-methyl-D-aspartate; NOS, nitric oxide synthase; RGC, retinal ganglion cell.

Several biochemical pathways and pharmacological targets have been investigated for potential neuroprotective roles in glaucoma. Glutamate is a prominent excitatory neurotransmitter in the retina, as well as in the brain, and glutamate receptors are recognized as a key pharmacological target of neuroprotective drugs. RGCs express a variety of glutamate receptors; under conditions of apoptosis, uncontrolled activation of glutamate receptors may result in large amounts being released into the extracellular region [6,12,25]. In these circumstances, glutamate acts as a neurotoxin, resulting in secondary neurodegeneration with loss of RGCs and damage to other retinal cells in continuity with them [6,11,12]. A proposed pathway of RGC death and presumed mechanisms of action of neuroprotective agents are shown in Figure 1. 

A therapeutic approach that directly targets neurons has the potential to prevent, block, delay or reduce glaucoma-related RGC death, in contrast to IOP lowering strategies that aim to indirectly provide neuroprotection by reducing the primary insult [11]. Potential neuroprotective agents may function either by increasing the survival stimuli and/or by hindering the neurotoxic signal, and, more generally, by targeting mechanisms responsible for triggering the apoptotic cascade [11]. For example, blocking the glutamate excitotoxicity cascade by interfering with glutamate release or inhibiting the glutamate receptor may be a promising neuroprotective strategy [11].

Other potential targets for neuroprotection include sites involved in promoting the survival of RGCs (including Müller cells, astrocytes, and the retinal microvasculature) and downregulating other mechanisms considered to act as triggers of the apoptotic cascade (oxidative stress, mitochondrial dysfunction, protein misfolding, and activation of astrocytes and microglia) [11,12]. 

## 4. Role of Citicoline as a Neuroprotective Agent

### 4.1. Role of Choline in Retinal Disorders

As noted, citicoline is metabolized to choline, a quaternary amine, and cytidine, a pyrimidine nucleoside, which are involved in brain phosphatidylcholine and phosphatidylethanolamine synthesis. Amongst the potential mechanisms involved in the pathogenesis of glaucoma, the perturbation of choline metabolism in advanced glaucoma and cortical cholinergic abnormalities have been implicated in glaucomatous degeneration [29]. Choline is an essential nutrient and a critical component of cell membranes, and thus it is crucial for cellular functioning [26,30]. As a precursor of the neurotransmitter acetylcholine, it is a basic component in the synthesis of the major neuronal membrane phospholipid, phosphatidylcholine, critical for ensuring structural integrity and facilitating both cell signaling and transport across the cell membrane [30]. Of relevance to this paper, cholinergic signaling is involved in neurocognitive function, visual neurophysiology, and interactions with neurons, including RGCs [31].

Nuclear magnetic resonance (NMR) spectroscopy has been used for the non-invasive systematic analysis of metabolic pathways that may identify disease-related biomarkers and lead to a better understanding of the pathophysiology of a disease state. In ophthalmology, NMR revealed a reduction in choline levels relative to creatinine (Cho:Cr) in the visual cortex in an experimental model of glaucoma [32] (induced hypertension in Sprague Dawley female rats), suggesting that dysfunction of the cholinergic system in the visual pathway may be a relevant component of the pathophysiological mechanisms involved in glaucoma. No other significant differences in metabolites were identified. These findings have been supported by studies in patients with glaucoma. In fact, using proton NMR spectroscopy, Zhang et al. found significant decreases in the ratios of Cho:Cr and, in addition, N-acetyl-aspartate (NAA):Cr in the geniculocalcarine and striate area of the occipital lobe in patients with primary glaucoma, pointing to an ongoing neurodegenerative process in the disease [33], potentially related to choline deficiency in brain tissues. 

Although further work is needed to confirm the importance of Cho:Cr in the visual cortex as a noninvasive marker for glaucoma, the dysfunction of the cholinergic system supports the importance of supplementation with a choline source, such as citicoline, in the glaucomatous disease. In this regard, supplementation with citicoline has shown significant benefits in patients with glaucoma [34,35,36,37,38,39,40,41,42,43].

The potential public health implications of deficiency of this essential nutrient, or of not receiving sufficient choline in the diet, have only recently begun to be examined [44]. Choline deficiency is thought to have an impact on diverse bodily systems, contributing to neurological and nervous system deficits and disorders of the liver, kidneys, pancreas, and muscle [30]. Although the significance of this for the development of glaucoma or other neurodegenerative diseases is not yet fully understood, it suggests a productive line of research. 

Good sources of dietary choline include eggs (in particular the yolk), organ meats (e.g., liver), legumes (e.g., soy), and wheat germ (a complete list is available at www.ars.usda.gov/ARSUserFiles/80400525/Data/Choline/Choln02.pdf). Choline intake tends to decrease with age, and there is a significant variation in individual dietary requirements for choline. Moreover, the average intakes in the population show considerable variation [30,44], and genetic polymorphisms appear to be responsible for the differences in the dietary requirement for choline [44]. Approximately 50% of the population may have genetic polymorphisms that increase choline requirements [44,45,46]. 

Using the definition of adequate intake level for choline set by the US Institute of Medicine in 1998, Vennemann et al. found that the average choline intake of European populations is below the recommended level [47], and Wallace et al. estimated that approximately 90% of the US population have a suboptimal intake of choline [48,49]. However, the definition of adequate intake for choline is now disputed, and it may not adequately reflect actual choline requirements. Therefore, it is difficult to draw conclusions about the adequacy of choline intake in most population groups [47]. Citicoline is a dietetic source of choline and cytidine and, in the event of increased choline requirements, supplementation with citicoline can be chosen as a safer and more effective option [50]. The tissue bioavailability of choline from citicoline—which exceeds 90% by both oral and parenteral routes [51,52]—appears to be superior to that of free choline [53]. Furthermore, there appears to be no data on the effects of choline supplementation on the physiology of the eye or in retinal disorders, in contrast to the published literature showing the bioavailability and benefits of citicoline supplementation in glaucoma and other ophthalmic diseases. Confirmation of citicoline deficiency, or increased citicoline requirements in patients at risk of developing glaucoma, represents an additional line of research that may further increase the understanding of the pathophysiology of the disease. 

### 4.2. Neuroprotective Properties of Citicoline

The pathophysiology of central neurodegenerative diseases may also involve, among other mechanisms, excitotoxicity, damage from oxidative stress, apoptotic effects, and inflammatory pathways. Citicoline is a neuroprotective compound with the potential to directly rescue RGCs or neutralize the detrimental effects of different toxic factors that have been studied. As for choline, citicoline is a precursor of the neurotransmitter acetylcholine and other neuronal membrane components, and acts as an intermediary in the synthesis of phosphatidylcholine [11,26,30,31]. Pharmaceutical grade citicoline, or cytidine 5′-diphosphocholine, is chemically identical to the naturally occurring metabolite [26,54]. 

Because the retina can be considered as part of the CNS, it has been suggested that glaucoma has similarities with other chronic central neurodegenerative diseases [6]. Moreover, the effect of citicoline supplementation on central functions has been investigated in several neurodegenerative diseases. In Parkinson’s disease, citicoline reduces the side effects of levodopa, and has a levodopa-saving effect, delaying the loss of efficacy [55,56]. 

There is evidence that citicoline has a positive effect on cognitive and behavioral disturbances in elderly patients with cognitive impairment associated with chronic cerebral disorders of the brain, such as Alzheimer’s disease [57,58,59]. Benefits in terms of memory, reaction time, attention, lucidity, concentration, behavior, global central functions, and general well-being have been reported. A Cochrane Database systematic review, which identified 14 trials that included 1051 patients with vascular cognitive impairment and senile dementia, confirmed the positive effect of citicoline on memory and behavior [60]. 

In ischemic stroke, some studies have shown that the administration of citicoline in addition to usual medical management for acute stroke may improve brain function and reduce post-stroke cognitive decline [61,62,63], in particular resulting in better outcomes in attention-executive functions and temporal orientation, compared with controls [64]. Prolonged administration of citicoline was safe and appeared to enhance neurogenesis and neural repair, particularly in older patients with less severe stroke not treated with recombinant tissue plasminogen activator [63].

Citicoline has been shown to have beneficial effects in a number of vision disorders. In amblyopia, citicoline, with or without patching, improved visual acuity [65,66,67,68,69] and contrast sensitivity and visually-evoked potentials (VEP) [69]. Similarly, citicoline improved visual acuity, VEP, and pattern electroretinogram (PERG) responses in patients with non-arteritic ischemic optic neuropathy (a human model of neurodegeneration) [70,71]. Furthermore, evidence from several preclinical and clinical studies suggest that citicoline has potential beneficial neuroprotective or neurorestorative activity in glaucoma.

### 4.3. Experimental Evidence

**Figure 2:**
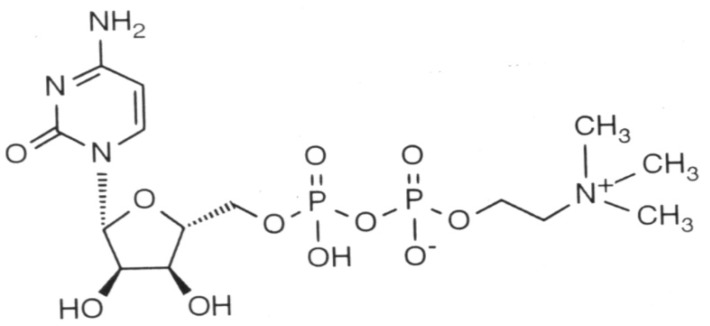
Chemical structure of cytidine 5′-diphosphocholine (citicoline), showing the cytidine molecule on the left and the diphosphocholine molecule on the right.

Figure 2 shows the chemical structure of citicoline. 

On absorption or after parenteral administration, citicoline undergoes rapid hydrolysis to cytidine-5′-monophosphate and phosphocholine, which are understood to be further dephosphorylated by phosphatases to choline and cytidine before crossing the blood–brain barrier, where choline is incorporated into the cytoplasmic and mitochondrial cell membranes and microsomal phospholipid fraction [25,72]. Uptake studies of radioactive-labeled citicoline by the intravenous (IV) or oral route in rodents confirm increasing levels of phospholipids, particularly phosphatidylcholine and sphingomyelin, in the brain in the first five hours following administration of citicoline, which continued to increase over the subsequent 48 h [72]. Concurrently, the cytidine fraction of citicoline is integrated into cytidine nucleotides and nucleic acids in the brain.

**Figure 3:**
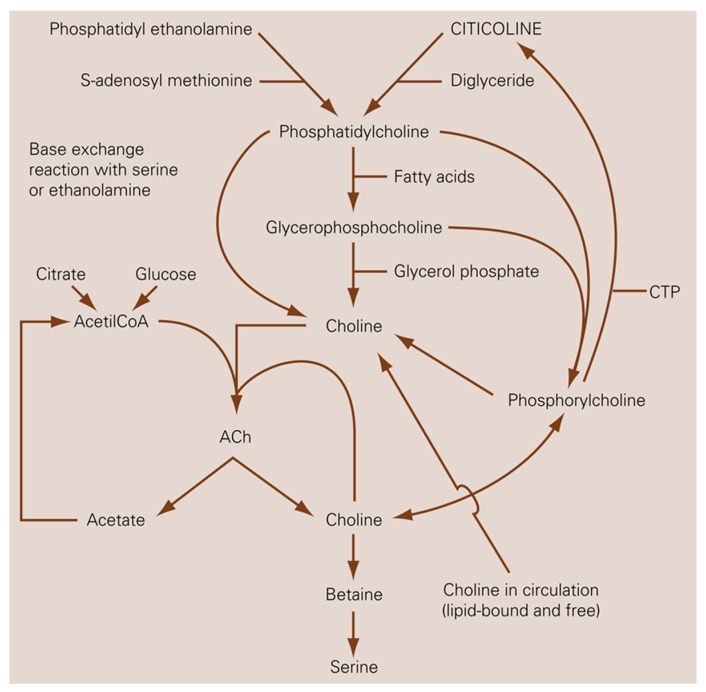
Relationship between citicoline and choline metabolism, cerebral phospholipids and acetylcholine. Ach, acetylcholine; CTP, phosphocholine cytidylyltransferase.

Experimentally, oral citicoline has been shown to activate the biosynthesis of phosphatidylcholine and other structural phospholipids of the neuronal membranes, increasing brain metabolism and enhancing the release of neurotransmitters in the CNS, including norepinephrine and dopamine [72]. As a precursor of acetylcholine (Figure 3), it participates in several relevant neurochemical processes and increases dopamine levels in the brain and retina [72]. 

Citicoline mediates neurodegenerative events by reducing glutamate excitotoxicity and oxidative stress, enhancing the release of neurotransmitters such as norepinephrine (noradrenaline) and dopamine, elevating neurotrophin levels, ameliorating axonal transport deficits, improving mitochondrial function including cardiolipin synthesis, restoring membrane integrity, and modulating insulin signaling [14,31].

Citicoline prevents ischemia-induced tissue accumulation of free fatty acids, resulting in an antiapoptotic and neuroprotective effect [25,72]. The antiapoptotic effect of citicoline has been postulated to be related to a mitochondrial-dependent cell death mechanism, together with the ability to support axon regeneration [73]. Specifically, in rodent retinal cultures and animal models of glaucoma, citicoline induced antiapoptotic effects in mitochondrial-dependent cell death, increased retinal levels of dopamine, counteracted retinal nerve fibers layer thinning, and promoted neurite regeneration (reviewed in Parisi et al., 2018 [52]). Furthermore, in vitro studies in retinal cells suggest that citicoline might have neuroprotective activity by blocking the excitotoxicity cascade leading to glutamate-mediated cell death [74,75] and decreasing both proapoptotic effects and synapse loss [76]. 

The effect of citicoline in preventing glutamate-mediated cell death was investigated in one study that used rat cerebellar granule cells pretreated with citicoline. Trypan blue exclusion and lactate dehydrogenase activity assays showed that citicoline caused a dose- and time-dependent reduction in glutamate-induced excitotoxicity [74]; citicoline reduced the number of apoptotic cells by over 80%. Cell death was more than halved when citicoline was added six days before glutamate excitotoxicity was induced, but less than 20% when citicoline was added concomitantly with glutamate. A second study examined whether citicoline has neuroprotective activity against kainic acid-induced retinal damage in the vitreous of rat eyes (kainic acid is an analog of glutamate). There was significant cell loss in the inner nuclear layer and the inner plexiform layer of the retinas on days 1, 3, and 7 after kainic acid injection [75]. At the same time points, no significant changes in retinal thickness or immunoreactivities of choline acetyltransferase and tyrosine hydroxylase in the retina were detected after treatment with citicoline, whereas immunoreactivities of choline acetyltransferase and tyrosine hydroxylase almost disappeared after seven days of kainic acid injection. However, prolonged treatment (7 days) with citicoline significantly attenuated the reduction in retinal thickness and immunoreactivity, supporting a neuroprotective effect on the retinal damage due to the glutamate-induced neurotoxicity.

The neuroprotective effect of citicoline against the toxic effects of prolonged hyperglycemia on the retina was examined by Zerbini et al. in a mouse model of type 1 diabetes [77]. After eight months of induced diabetes, there was a reduction in thickness of both the choroid and the retinal layers, including the retinal nerve fiber layer, ganglion cell complex, and the ganglion cells/inner plexiform layer, in diabetic versus control mice. However, there was no significant thinning in the corresponding retinal layers in citicoline-treated diabetic mice [77], suggesting a neuroprotective action.

A triad summarizing the biochemical and biological activities of citicoline on neurodegeneration has been proposed [31]: 

(1) Citicoline protects undamaged axons—neuroprotective activity (e.g., reduction in glutamate-induced neurotoxicity).

(2) Citicoline rescues partially damaged neurons—neurorestorative activity (e.g., membrane reintegration of damaged neurons). 

(3) Evidence from in vitro studies supports the ability of citicoline to regenerate neuronal cells—regenerative activity (e.g., biosynthesis of structural components of the cell membrane).

The rapid hydrolysis of citicoline to cholinergic and pyrimidinergic catabolites available for use by diverse metabolic pathways contributes to its lack of acute and chronic toxicity, which has been confirmed in numerous studies in animals and humans [52,72]. In fact, the median lethal dose (LD50) of IV citicoline in animal models (4150 to 4600 mg/kg) is approximately 44 times higher than that of an equivalent IV dose of choline [52], suggesting that the administration of citicoline has potential metabolic consequences dissimilar to those of exogenous choline. In the same way, the theoretical oral LD50 of citicoline has been estimated to be in the order of 20,000–30,000 mg/kg, whereas the therapeutic dose range in humans is between 500 and 2000 mg/kg [52]. As reviewed by Secades in 2016, with the exception of minor digestive intolerance and occasional excitability or restlessness in the first few days of treatment, the safety and tolerability of citicoline is rated as excellent and free of serious adverse events in healthy individuals and in patients of varying ages and with different concomitant disorders [72]. However, while the safety of citicoline is largely agreed on, it is also important to note that non-compliance in the daily pharmacological therapy for IOP may result in spikes that lead to a worsening of retinal sensitivity and thinning of the retinal nerve fiber layer.

Support for a neuroprotective role for citicoline in glaucomatous neurodegeneration have come from a number of in vitro and in vivo studies, using retinal cell cultures and animal models of glaucoma, as well as from clinical trials that have demonstrated that citicoline has the potential to ameliorate glaucomatous damage or vision loss (Table 1 and Table 2). 

The neuromodulator effects of citicoline in protecting neuronal cells such as RGCs have been demonstrated in the rabbit retina [78]. Evaluation of retinal catecholamine levels showed that animals treated with citicoline had increased dopamine levels compared with animals treated with vehicle. Citicoline stimulated the density of RGC connected with the superior colliculus in an animal model of optic nerve crush, together with evidence of an antiapoptotic effect, mediated by increased expression of the antiapoptotic protein, Bcl-2 [79].

In addition, various studies have confirmed the favorable safety characteristics of citicoline in in vivo models of retinal neurodegeneration (reviewed in Roberti et al., 2015 [11]).

Table 1 provides a summary of in vitro and in vivo trials of citicoline in models of retinal damage, showing neuroprotective and neuroregenerative effects.

### 4.4. Evidence from Clinical Studies

Oral and intramuscular (IM) citicoline has been shown to improve retinal function and neural conduction along visual pathways in patients with glaucoma [38,41]. The use of citicoline to treat POAG was first reported in 1989 by Pecori Giraldi et al., who described favorable neurotrophic effects on the visual fields of patients treated with IM citicoline [40]. All patients in their study had glaucomatous perimetric defects despite well-controlled IOP. Treatment with IM citicoline 1 g daily for 10 days significantly improved perimetric conditions in the majority of eyes, based on reduction in the scotomatous area and a decrease in the mean perimetric defect. The beneficial effects were treatment-dependent and maintained for at least three months; the favorable effects were again obtained on retreatment [40], suggesting that citicoline administration must be repeated periodically for optimal effect. This was the first confirmation that citicoline supplementation could positively modify glaucomatous optic nerve damage. In a follow-on study by the same researchers, the protective effects of repeated cycles of citicoline therapy (1 g IM for 15 days, every 6 months) in preventing the progression of perimetric deficits were maintained over 10 years of follow-up [43]. Citicoline reduced the visual field worsening fraction (≤10% vs. ≥50% in treated patients vs. controls, *p* = 0.007), and significantly improved retinal sensitivity (*p* < 0.05), which remained stable throughout treatment [43].

Other placebo-controlled studies have shown retinal and cortical responses in patients with glaucoma treated with two months of IM citicoline, with further enhancements of visual function when the treatment was repeated after a washout period [39]. IOP was controlled by topical β-blockers in both treatment groups. Benefits were maintained after eight years of follow-up when these patients continued treatment with IM citicoline in cycles of 2 months of treatment followed by 4 months of wash-out [36]. Improvements in VEP and PERG parameters achieved with the initial treatment cycle declined during the wash-out period, without returning to baseline levels, and further improved with subsequent cycles (*p* < 0.01 vs. baseline and placebo). These data suggest the potential for citicoline to stabilize or improve glaucomatous visual function in conjunction with conventional ocular antihypertensive therapy.

However, IM therapy is unlikely to be a preferred route of administration for patients with a chronic disease such as glaucoma. Oral administration of citicoline, which appears to have minimal side effects, is considered preferable as it improves compliance while maintaining the central benefit not provided by topical administration [6,25], and may be better than either alternative route of administration in slowing or preventing central neural degeneration. 

A clinical study by Rejdak et al. was the first to investigate the efficacy of citicoline by oral administration; patients were treated with tablets containing 500 mg of the active ingredient, given twice-daily for two bi-weekly courses [41]. Citicoline normalized VEP compared with baseline in 62% of glaucomatous eyes, with VEP latency reduced from 123.5 ms to 119.9 ms (*p* < 0.001) and VEP amplitude increased from 6.56 μV to 7.88 μV (*p* < 0.05).

A study by Parisi et al. that compared oral (1600 mg/day) and IM (1000 mg/day) citicoline for two 60-day treatment periods in glaucoma patients with moderate visual defects found objectively-evaluated improvements in retinal function and neural conduction along visual pathways for both routes of administration [38], confirming the efficacy of citicoline by the preferred oral route. Partial regression occurred after two, 120-day washout periods, suggesting that continued supplementation is necessary to maintain the potential neuroprotective effects of citicoline in glaucoma. Extension of the treatment for up to eight years duration stabilized or further improved the glaucomatous visual dysfunction [38].

Oral citicoline appeared to have neuroprotective activity in patients with chronic POAG [34]. Patients with chronic open-angle glaucoma were treated with oral citicoline 500 mg daily or placebo for three cycles of 60 days separated by washout periods of 30 days. The mean VEP amplitude increased by the end of each treatment cycle and moderately regressed by the end of each washout period. Retinocortical time also significantly reduced during citicoline treatment. VEP amplitude and retinocortical time remained unchanged in the placebo group [34].

Other evidence for the long-term benefits of citicoline by oral administration, has been shown in patients with progressing POAG despite controlled IOP [35,80]. Oral citicoline 500 mg daily in cycles of 4-month periods separated by 2-month wash-out significantly slowed the rate of glaucomatous progression during the 2-year study. At baseline, all patients had a progressive decrease (at least −1 dB/year) of visual field parameters within the prior three years, and an IOP controlled to <18 mmHg. The rate of glaucomatous progression reduced significantly during treatment to a mean of −0.15 ± 0.3 dB/year at the study end (*p* = 0.01). The efficacy of oral citicoline in slowing the progression of glaucoma was also endorsed by the 2-year results of a study that evaluated both functional (standard automated white-on-white perimetry (SAP)) and morphological (optical coherence tomography (OTC)) parameters in 60 patients with POAG glaucoma [80]. Of interest, the effects developed slowly over time, and became significantly different compared with controls only after more than a year of treatment, suggesting that extended therapy is necessary to achieve clinically meaningful results. At 18 months, the mean deviation of SAP mean values was significantly higher in the citicoline group, compared with controls, and remained stable over time, whereas values continued to decrease in the control group. Retinal nerve fiber layer thickness and ganglion cell complex thickness also significantly improved with citicoline therapy.

Citicoline administered by topical route as eye drops has also been shown to improve retinal function and neural conduction along the visual pathway [37,42,81]. After citicoline topical treatment, enhancement of retinal bioelectrical responses and bioelectrical activity of the visual cortex were demonstrated by an increase in PERG amplitude and shortening of VEP implicit time, respectively.” The effects on visual parameters were similar to those observed with oral citicoline. However, eye drop formulations require penetration enhancers to ensure sufficient citicoline levels in the vitreous, which increases the likelihood of adverse events, in contrast to orally administered citicoline, in which side effects are minimal or absent [6]. Oral administration of citicoline also provides a central benefit that cannot be given by the topical form. Furthermore, adding another medication in eye drop formulation is not ideal and may compromise compliance.

A summary of clinical trials of citicoline in patients with glaucoma is presented in Table 2. The data reported in the table support the hypothesis that citicoline has neuroprotective, neurorestorative, and neuroregenerative effects on the visual system.

As discussed, citicoline displays negligible toxicity [6,11,14,25], and its lack of acute and chronic adverse events contributed to its authorization as a food supplement in the European Union (EU) and the USA. In the EU, the approval for citicoline is as a novel food ingredient in food supplements and in dietary foods for special medical purposes (Decision 2014/423/EU). Furthermore, citicoline has been approved by the Italian Ministry of Health as a dietary food supplement for special medical purposes in glaucoma patients with respect to its benefits shown in these subjects.

## 5. Discussion

Elevated IOP remains a key modifiable risk factor and an important therapeutic target in glaucoma, one of the most common neurodegenerative optic pathologies that leads to irreversible blindness. However, risk factors other than IOP are recognized to be involved in modulating the disease course of this complex and multifactorial disease, the pathophysiology of which is not completely understood. Ocular hypotensive therapy alone is not sufficient to adequately manage the RGC loss and progressive reduction in visual fields characteristic of glaucomatous disease [2], and the hypothesis that factors other than IOP intervene in the pathogenesis of glaucoma is supported by analysis of long-term trends in glaucoma-related blindness in population-based cohort studies [8,9]. Therefore, in addition to conventional therapy, there is a need for additional treatment modalities for glaucomatous neuropathy that address glaucoma-induced damage by treating the entire central visual pathway and promoting neuroprotection and neuroregeneration. 

Early intervention is vital to minimize ongoing damage to the visual field that will be present due to the loss of RGCs already existing at the diagnosis of glaucoma. In this respect, several therapeutic strategies have been investigated, among which the naturally occurring compound and novel food ingredient for special medical purposes, citicoline, has shown potential beneficial neuroprotective properties in glaucoma and other vision disorders in studies representing decades of research data. 

As is the case with the pathophysiology of central neurodegenerative diseases, glaucomatous mechanisms involve glutamate excitotoxicity, damage from oxidative stress, apoptotic effects, and inflammatory pathways [11]. Citicoline has shown activity in the treatment of patients with cognitive and behavioral disturbances related to chronic cerebral disorders, and in stroke, Alzheimer’s disease, Parkinson’s disease, and traumatic brain injury [14,25,31]. Indeed, the same central benefits studied in patients with neurodegenerative diseases are also relevant to patients with glaucoma, the majority of whom are elderly, with the potential to improve not only visual function but also cognitive function. In addition to data from experimental studies in these diverse neurodegenerative diseases, and increasingly in patients with glaucoma, there is evidence that citicoline may have neuroenhancement, neuroprotective, and neurorestorative activity [31]. Indeed, citicoline appears to be a compound with the potential to directly rescue RGCs by blocking the excitotoxicity cascade leading to glutamate-mediated cell death while reducing oxidative damage and improving mitochondrial function. 

The mechanisms by which citicoline achieves its beneficial effects include its role as a precursor of the neurotransmitter acetylcholine and other neuronal membrane components, leading to enhanced release of neurotransmitters in the CNS, and its action as an intermediary in the synthesis of phosphatidylcholine, essential for the membrane integrity of RGCs [30]. In this way, citicoline may prevent the deterioration of membranes in neurons and inhibit the apoptosis that occurs in neurodegenerative processes.

The potential effects of citicoline in protecting RGCs from neurodegeneration, improving deteriorating retinal function and neural conduction along visual pathways, promoting reversal of RGC damage, supporting axon regeneration, and protecting undamaged axons in glaucoma are promising. Further studies in animal models and clinical trials should be conducted to provide additional insights into the effects of citicoline in glaucomatous disease, and to investigate whether citicoline supplementation has as a broader preventive application in a more general population.

## 6. Conclusions

Although direct evidence of citicoline deficiency in patients with glaucomatous disease is currently lacking, the beneficial effects of citicoline supplementation in glaucoma and its registration as a dietary food supplement for special medical purposes suggest that citicoline could be considered a promising molecule for neuroprotective strategies in glaucoma. This is supported by the literature of experimental and human studies in neurodegenerative diseases and in other visual disorders where functional and/or morphological changes of RGCs occurs.

## Figures and Tables

**Table 1:** Summary of experimental studies evaluating the effect of citicoline on retinal ganglion cells in vitro and in animal models of glaucoma.

Authors	Study Design	Citicoline Concentration	Outcome Measures
Retinal cell cultures			
Oshitari et al., 2002 [73]	Cultured mouse retina	0.01–10 μM cultured for 9 days	TUNEL staining and assessment of the number of regenerating neurites on damaged RGCs
Matteucci et al., 2014 [76]	Cultured rat retinas	10, 100 and 1000 μM for 96 h and 24 h before glutamate-induced excitotoxic insult and high glucose-promoted neuronal cell damage	Apoptotic analysis and immunostaining and morphometric analysis of glutamate- and hyperglycemia-induced RGC damage
Animal models			
Rejdak et al., 2002 [78]	Case-control in albino rabbits	IP administration of 50 mg/kg twice day	Retinal catecholamine levels
Park et al., 2005 [75]	Case-control in adult Sprague-Dawley rats	IP administration of 500 mg/kg twice for 1, 3, and 7 days after KA injection	Retinal layer thickness and expression of ChAT and TH after KA-induced retinal damage
Schuettauf et al., 2006 [79]	Case-control in adult Brown Norway rats	IP administration of 1g/kg/daily and 300 mg/kg/daily	Density of RGCs and expression of the antiapoptotic protein Bcl-2
Zerbini et al., 2015 [77]	Mouse model of type 1 diabetes	Topical application of 2% eye drops	Retinal layer thickness and choroidal thickness

ChAT, choline acetyltransferase; IP, intraperitoneal; KA, kainic acid (glutamate analog); RGC, retinal ganglion cell; TH, tyrosine hydroxylase; TUNEL, terminal deoxynucleotidyl transferase-mediated dUTP-biotin nick end labeling.

**Table 2:** Summary of clinical studies evaluating the effects of citicoline in glaucoma patients.

Authors	Study Design	Study Population	Administration and Dosage	Treatment Schedule	Follow-up	Outcome Measures
Pecori Giraldi et al., 1989 [40]	Cohort	OAG (*n* = 30)	IM 1 g/day	10 days	3 months	Reduction in the scotomatous area (computerized central perimetry) and decrease in mean defect (automated perimetry)
Parisi et al., 1999 [39]	Double-blind placebo controlled	OAG (*n* = 40) −3 dB > MD < −6 dB	IM 1 g/day	2 cycles of 60 days with 120-day washout period	360 days	VEP and PERG parameters
Virno et al., 2000 [43]	Case-control	OAG (*n* = 23)	IM 1 g/day	15 days treatment repeated every 6 months for 20 cycles	10 years	Visual field worsening (increase in non-perception area >500 mm^2^)
Rejdak et al., 2003 [41]	Cohort	OAG (*n* = 21 eyes)	Oral 1 g/day	14 days 2 days of washout (2 cycles)	56 days	VEP parameters
Parisi V, 2005 [36]	Case-control	OAG (*n* = 30) −3 dB > MD < −6 dB	IM 1 g/day	60-day cycles with 120 days of washout (14 cycles)	8 years	VEP and PERG parameters
Parisi et al., 2008 [38]	Case-control	OAG (*n* = 60) −2 dB > MD < −14 dB	IM 1 g/day Oral 1600 mg/day	60 days 120 days of washout (2 cycles)	360 days	VEP and PERG parameters
Ottobelli et al., 2013 [35]	Retrospective cohort	Progressing OAG (*n* = 41)	Oral 500 mg/day	120 days 60 days of washout (4 cycles)	2 years	Rate of visual field progression
Roberti et al., 2014 [42]	Case-control	OAG (*n* = 34) −3 dB > MD < −12 dB	Intraocular (topical eye drops) 3 drops/day	60 days	90 days	VEP and PERG parameters
Parisi et al., 2015 [37]	Case-control	OAG (*n* = 56) MD > −10 dB	Intraocular (topical eye drops) 3 drops/day	120 days 60 days of washout	180 days	VEP and PERG parameters
Lanza et al., 2019 [80]	Case-control	OAG (*n* = 60) MD −6.51 dB	Oral 500 mg/day	120 days 60 days of washout (4 cycles)	2 years	SAP and OCT parameters

IM, intramuscular; MD, mean deviation; OAG, open-angle glaucoma; OCT, optical coherence tomography (OCT); PERG, pattern electroretinogram; SAP, standard automated white-on-white perimetry; VEP, visual evoked potential.
